# Structural study of bioisosteric derivatives of 5-(1*H*-indol-3-yl)-benzotriazole and their ability to form chalcogen bonds

**DOI:** 10.1107/S2056989022002948

**Published:** 2022-03-22

**Authors:** Manon Mirgaux, Tanguy Scaillet, Arina Kozlova, Nikolay Tumanov, Raphaël Frederick, Laurie Bodart, Johan Wouters

**Affiliations:** a Namur Institute of Structured Matter (NISM), Namur Research Institute for Life Science (NARILIS), Department of Chemistry, Laboratoire de Chimie Biologique Structurale (CBS) University of Namur (UNamur), 61 Rue de Bruxelles, 5000, Namur, Belgium; b Louvain Drug Research Institute (LDRI), Université Catholique de Louvain (UCLouvain), Brussels B-1200, Belgium

**Keywords:** crystal structure, bioisosterism, chalcogen, chalcogen inter­action, tryptophan-2,3-di­oxy­genase inhibitors, X-ray crystallography

## Abstract

Recently, inter­est in the isosteric replacement of a nitro­gen atom to selenium, sulfur or oxygen atoms has been highlighted in the design of potential inhibitors for cancer research. In this context, the structures of 5-(1*H*-indol-3-yl)-2,1,3-benzotriazole derivatives [5-(1*H*-indol-3-yl)-2,1,3-benzo­thia­diazole (bS, C_14_H_9_N_3_S) and 5-(1*H*-indol-3-yl)-2,1,3-benzoxa­diazole (bO, C_14_H_9_N_3_O)], as well as a synthesis inter­mediate of the selenated bioisostere [5-[1-(benzensulfon­yl)-1*H*-indol-3-yl]-2,1,3-benzoselena­diazole (p-bSe, C_20_H_13_N_3_O_2_SSe)] were determined using single-crystal X-ray diffraction (SCXRD) analyses. Despite being analogues, different crystal packing, torsion angles and supra­molecular features were observed, depending on the substitution of the central atoms of the benzotriazole. In particular, chalcogen inter­actions were described in the case of p-bSe and not in the bS and bO derivatives. An investigation by *ab initio* computational methods was therefore conducted to understand the effect of the substitution on the ability to form chalcogen bonds and the flexibility of the compounds.

## Abstract 2

Recently, inter­est in the isosteric replacement of a nitro­gen atom to selenium, sulfur or oxygen atoms has been highlighted in the design of potential inhibitors for cancer research. In this context, the structures of 5-(1*H*-indol-3-yl)-2,1,3-benzotriazole derivatives [5-(1*H*-indol-3-yl)-2,1,3-benzo­thia­diazole (bS, C_14_H_9_N_3_S) and 5-(1*H*-indol-3-yl)-2,1,3-benzoxa­diazole (bO, C_14_H_9_N_3_O)], as well as a synthesis inter­mediate of the selenated bioisostere [5-[1-(benzensulfon­yl)-1*H*-indol-3-yl]-2,1,3-benzoselena­diazole (p-bSe, C_20_H_13_N_3_O_2_SSe)] were determined using single-crystal X-ray diffraction (SCXRD) analyses.

## Chemical context

**Figure 1:**
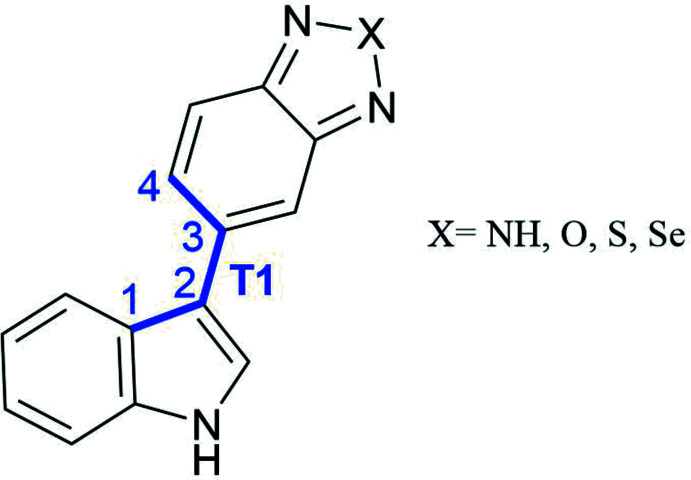
Structures of bioisosteres of 5-(1*H*-indol-3-yl)-benzotriazole with their torsion angle. *X* = NH, O, S, Se.

Isosteric replacement is a common strategy in drug design to modulate the physicochemical properties of potential inhibitors. In 2021, Kozlova and co-workers (Kozlova *et al.*, 2021*a* ▸) highlighted a series of bioisosteric derivatives acting as potential new inhibitors of the protein hTDO2, a therapeutic target in cancer research. These new mol­ecules differ in the replacement of the central atom of benzotriazole by an oxygen, sulfur or selenium atom (Fig. 1 ▸). At this time, these inhibitors have not yet been crystallized or structurally characterized. In this context, the present work provides a structural characterization of the inhibitors described by Kozlova *et al.* (2021*b* ▸) completed by *ab initio* calculations for their conformational characterization.

The contribution of an oxygen, a sulfur or a selenium atom instead of a nitro­gen affects the ability of these inhibitors to participate in the formation of chalcogen bonds (Vogel *et al.*, 2019 ▸). In particular, in these compounds, oxygen, sulfur and selenium atoms could act as chalcogen-bond donors. In recent years, the importance of chalcogen bonds in the stability and folding of proteins as well as their inter­action with ligands has been highlighted by numerous investigations (Newberry & Raines, 2019 ▸; Kříž *et al.*, 2018 ▸; Iwaoka *et al.*, 2001 ▸; Iwaoka & Babe, 2015 ▸; Burling & Goldstein, 1992 ▸). In this article, the potential ability of the compounds to inter­act with aromatic groups, by chalcogen–π inter­actions (Aakeroy *et al.*, 2019 ▸), was revealed by the crystallization of 5-[1-(benzensulfon­yl)-1*H*-indol-3-yl] −2,1,3-benzoselena­diazole. Therefore, the effect of bioisosteric replacement on the ability to form chalcogen bonds has been studied by *ab initio* calculated electrostatic potential maps. This inter­esting series could be the starting point for the study of the effect of chalcogen inter­action on protein stability and affinity. 
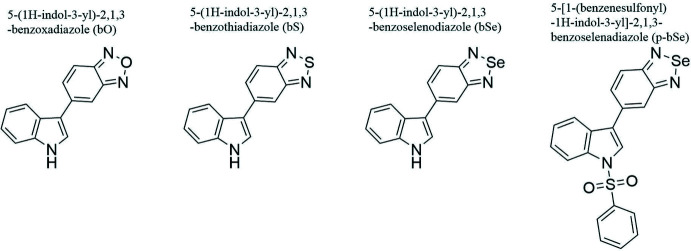


## Structural commentary

The compounds investigated in this study were kindly provided by the team of Raphaël Frédérick (UCLouvain, Belgium). Crystallization assays were performed, by slow evaporation at room temperature (293–298 K), in four different solvents [tetra­hydro­furan (THF), chloro­form, di­chloro­methane and *N*,*N*-di­methyl­formamide (DMF)]. Crystals of 5-(1*H*-indol-3-yl)-2,1,3-benzoxa­diazole (bO) and of 5-(1*H*-indol-3-yl)-2,1,3-benzo­thia­diazole (bS) were obtained from chloro­form. Despite numerous attempts, we were not able to crystallize the compound 5-(1*H*-indol-3-yl)-2,1,3-benzoselena­diazole (bSe). However, crystals of a synthesis inter­mediate – 5-[1-(benzensulfon­yl)-1*H*-indol-3-yl]-2,1,3-benzo­selena­diazole (p-bSe) – were obtained in THF.

**Figure 2:**
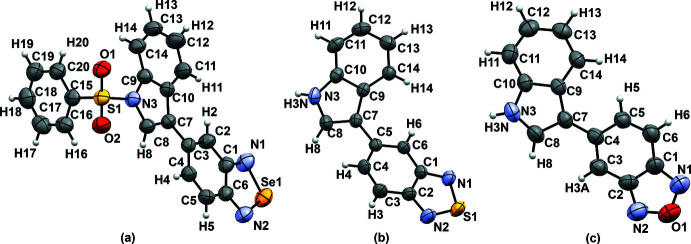
Ellipsoid plots with atom labeling for (*a*) p-bSe (*b*) bS and (*c*) bO. Displacement ellipsoids are drawn at the 50% probability level.

**Figure 3:**
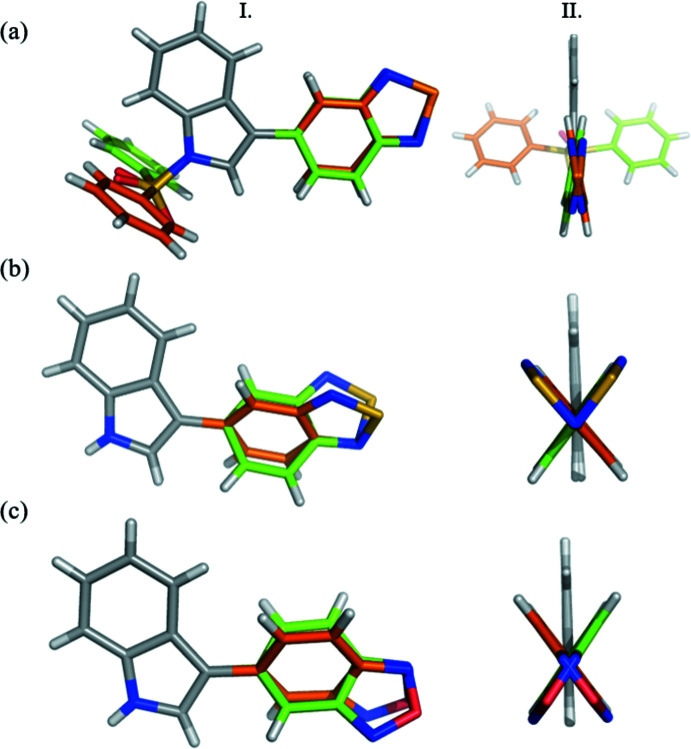
Two perpendicular views (I. and II.) of the two mirror images observed in the crystal packings for (*a*) p-bSe (*b*) bS and (*c*) bO. Superposition of the mirror images with respect to the indole group. Torsion angles are ±168.3 (2)° for p-bSe, ±36.9 (2)° for bS and ±146.7 (2)° for bO.

5-[1-(Benzensulfon­yl)-1*H*-indol-3-yl]-2,1,3-benzoselena­dia­zole (p-bSe) crystallized in space group *P* 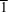 with one mol­ecule of p-bSe in the asymmetric unit [Fig. 2 ▸(*a*)]. Inter­estingly, the mol­ecule adopts an almost planar dihedral angle [−168.3 (2)°] between the indole and benzoselena­diazole ring (Fig. 3 ▸). 5-(1*H*-indol-3-yl)-2,1,3-benzo­thia­diazole (bS) and 5-(1*H*-indol-3-yl)-2,1,3-benzoxa­diazole (bO) crystallized in space group *Pbca* [Fig. 2 ▸(*b*) and (*c*)]. The asymmetric units contain one mol­ecule of bS or bO without disorder. In the three structures, two mirror images are observed in the crystal packing with a torsion angle of ±168.3 (2)° for p-bSe, ±36.9 (2)° for bS and ±146.7 (2)° for bO between the two aromatic parts of the mol­ecules (Fig. 3 ▸). The isosteric replacement does not change significantly the planarity of the benzotriazole ring (r.m.s. deviation from planarity: 0.013 Å for p-bSe, 0.006 Å for bS and bO) or the indole ring (0.010 Å for p-bSe, 0.025 Å for bS and 0.011 Å for bO).

## Supra­molecular features

**Figure 4:**
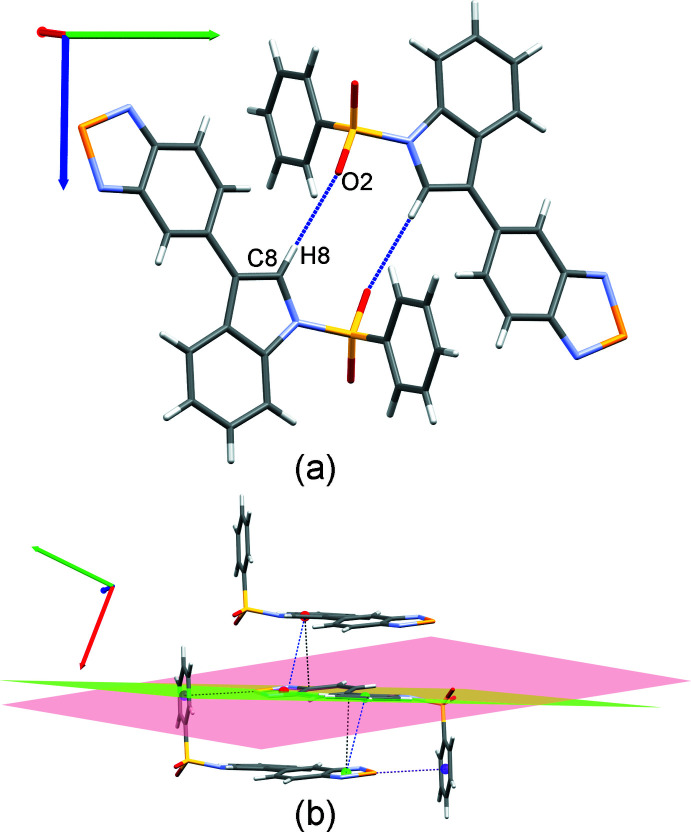
Supra­molecular organization of p-bSe: (*a*) hydrogen-bond inter­actions; (*b*) π-stacking inter­action between the benzoselena­diazole group and the indole groups (centroids in red: Se/N1/C1/C6/N2 and N3/C7–C10 and centroids in green: Se/N1/C1/C6/N2 and C9–C14) as well as chalcogen–π inter­actions (in purple).

In the structure of p-bSe, a synthesis inter­mediate of the selenated bioisostere of 5-(1*H*-indol-3-yl)benzotriazole, the benzene­sulfonyl contributes to the stabilization of the crystal packing through weak hydrogen bonds [Table 1 ▸, Fig. 4 ▸(*a*)] and chalcogen–π inter­actions. π-stacking inter­actions are observed between the selena­diazole and indole groups [centroid (Se/N1/C1/C6/N2)⋯centroid (N3/C7–C10) distance of 3.732 (2) Å, perpendicular distance of 3.587 (1) Å and horizontal displacement of 1.506 Å, Fig. 4 ▸(*b*)]. A second π-stacking inter­action is observed between the selena­diazole group (Se/N1/C1/C6/N2) and the indole group (C9–C14) [centroid⋯centroid distance of 3.915 (2) Å, perpendicular distance of 3.646 (1) Å and horizontal displacement of 1.331 Å, Fig. 4 ▸(*b*)]. A chalcogen–π inter­action between Se and the benzensulfonyl group (C15–C20) is also involved in crystal-packing stabilization [Se⋯centroid distance of 3.388 (1) Å and N2—Se⋯centroid angle of 159.83 (8)°, Fig. 4 ▸(*b*)]. The presence of the protecting group (benzene­sulfon­yl) could explain the crystallization of the p-bSe compound with respect to the bSe compound. Indeed, in p-bSe, the orientation of the protecting group is ideal for allowing a chalcogen–π inter­action whereas this type of inter­actions would be more difficult to set up in bSe.

**Figure 5:**
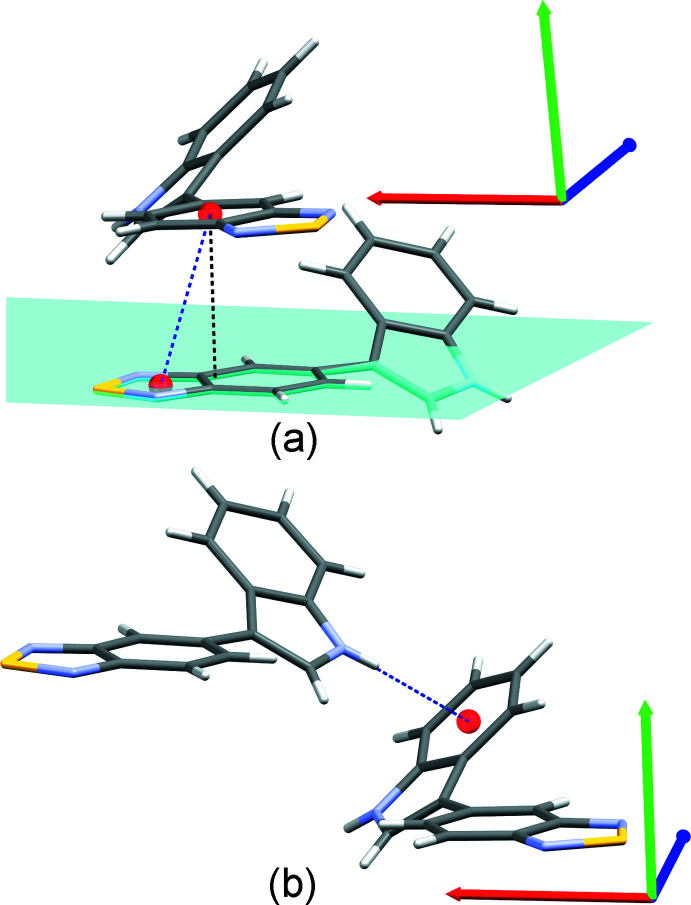
Supra­molecular organization of bS. (*a*) π-stacking inter­action between the benzo­thia­diazole groups and (*b*) N—H⋯π inter­action between two indole groups.

In the structure of compound bS, π-inter­actions stabilize the crystal packing. π-stacking is observed between benzo­thia­diazole groups [centroid (C1–C6)⋯centroid (S1/N1/C1/C2/N2) distance of 3.689 (1) Å, perpendicular distance of 3.4989 (7) Å and horizontal displacement of 1.326 Å, Fig. 5 ▸(*a*)]. An N—H⋯π inter­action is also observed between indole groups [N3⋯centroid (C9–C14) distance of 3.345 (2) Å, H3*N*⋯centroid distance of 2.57 (2) Å and N3—H3*N*⋯centroid angle of 169 (2)°, Fig. 5 ▸(*b*)]. In this structure, no chalcogen inter­action involving the sulfur atom is observed.

**Figure 6:**
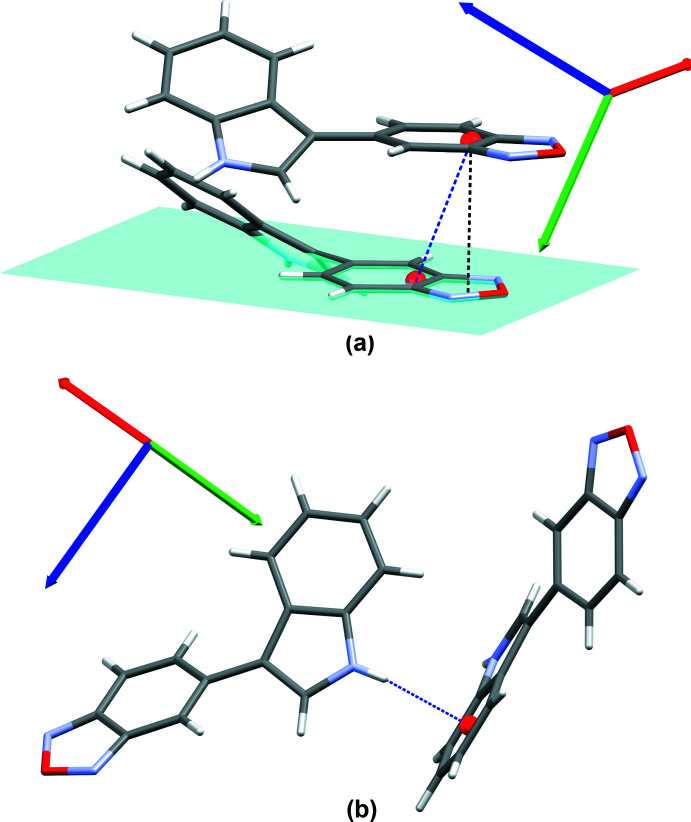
Supra­molecular organization of bO. (*a*) π-stacking inter­action between the benzoxa­diazole groups and (*b*) N—H⋯π inter­action between two indole groups

The crystal packing of bO is stabilized through π-inter­actions. π-stacking is observed between benzoxa­diazole groups [centroid (O1/N1/C1–C6/N2)⋯centroid (O1/N1/C1–C6/N2) distance of 3.893 (1) Å, perpendicular distance of 3.5469 (8) Å and horizontal displacement of 1.570 Å, Fig. 6 ▸(*a*)]. An N—H⋯π inter­action is also observed between indole groups [N3⋯centroid (C9–C14) distance of 3.226 (2) Å, H3*N*⋯centroid distance of 2.57 (2) Å and N3—H3*N*⋯centroid angle of 138 (2)°, Fig. 6 ▸(*b*)]. No chalcogen inter­action is observed.

## Quantum *ab initio* studies of the bioisosteric substitution effect

**Figure 7:**
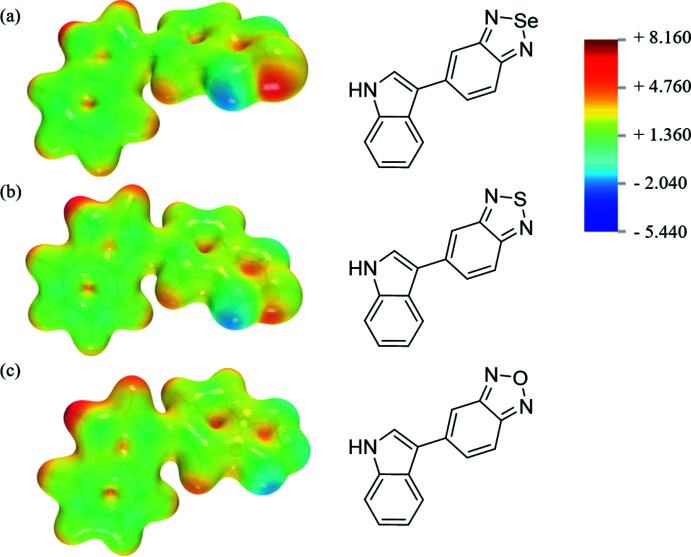
Computed electrostatic potential (EPS) surfaces and associated mol­ecular structures (*a*) bSe (*b*) bS (*c*) bO. The EPS color scale ranges from +8.160 volt to −5.440 volt.

**Figure 8:**
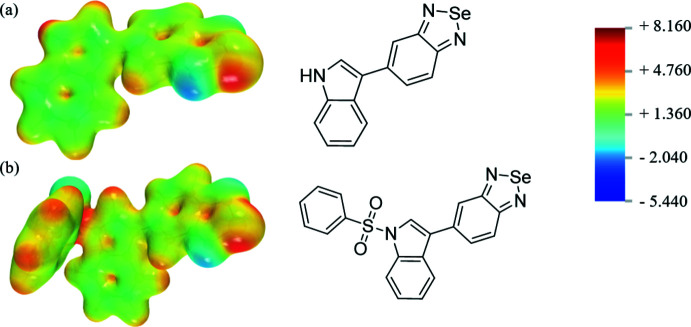
Computed electrostatic potential (EPS) surfaces and associated mol­ecular structures (*a*) bSe (*b*) p-bSe. The EPS color scale ranges from +8.160 volts to −5.440 volts.

As mentioned previously, the different derivatives vary mainly in their ability to inter­act through chalcogen bonds. In order to characterize these differences in depth, quantum mechanics studies have been conducted. First, the presence of a σ-hole in the electron density was studied by means of electrostatic maps. Analysis indicates that the oxygen in benzoxa­diazole [Fig. 7 ▸(*c*)] has a weakly positive environment. The σ-hole formation is enhanced by substitution of the central atom with sulfur [Fig. 7 ▸(*b*)] and selenium [Fig. 7 ▸(*a*)], with selenium having the most positive environment. The bioisosteric series thus has different characteristics in terms of the ability to form chalcogen bonds, with the selenium compound being the best chaclogen-bond donor in this bioisosteric series of mol­ecules. These results may explain why chalcogen bonds are observed only in the supra­molecular organization of the p-bSe mol­ecule. The donor character of the selenium atom is not affected by the protected group [Fig. 8 ▸(*a*) and (*b*)]. The difficulty in crystallizing bSe (while p-bSe crystallized readily in THF) could be explained by the absence of the protecting group (benzene­sulfon­yl) in bSe. Indeed, the benzene­sulfonyl group in p-bSe is electron-rich and acts as a well-oriented chalcogen-bond acceptor in p-bSe.

**Figure 9:**
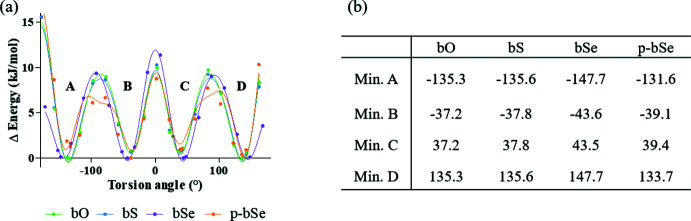
Conformational scans and associated torsion angles calculated with *Gaussian16a* (ωB97XD, 6–31+G*).

Secondly, in order to determine the effect of the substitution on the flexibility of the derivatives, conformational scans were performed around the torsion angle formed between the indole ring and the benzo­diazole part (*T*1). As presented in Fig. 9 ▸, bO and bS are characterized by a very similar Δ*E* energy profile associated with the rotation around *T*1. For all three mol­ecules (bO, bS and bSe), four minima are observed for each mol­ecule, with symmetry on each side of the planar mol­ecule. The energy transitions are low (maximum 15 kJ mol^−1^) and the mol­ecules are flexible. Moreover, the *T*1 torsion angles observed in the crystal structures of bS [±36.9 (2)°] and bO [±146.7 (2)°] are consistent with the energy minima determined by *ab initio* calculations with a relative deviation lower than 10%. Although the bioisosteric character of the flexibility is retained, two differences are observed between the bSe mol­ecule and the bS and bO mol­ecules. The first one is the energy at a torsion angle of 180°, which is lower for the mol­ecule of bSe while it is slightly higher at 0°. The second one is a small shift observed between the angle associated with the energy minima of the bSe mol­ecule and the bS and bO mol­ecules.

The same calculations were performed for the protected mol­ecule (p-bSe). The energy profile associated with the rotation around *T*1 in p-bSe is similar to those determined for bS and bO. There is a small shift of the values of the angle corresponding to the energy minimum with respect to bSe. This shift may be due to the protecting group that causes steric hindrance in p-bSe. The energies corresponding to minima *A* and *C* are higher than the energies for minima *B* and *D*, involving two local minima and a preference in the conformers. The maxima of the energies between *A*/*B* and *C*/*D* are also lower than in the case of the other compounds, supporting this hypothesis. In the case of p-bSe, the torsion angle observed in the crystallographic structure [±168.3 (2)°] corresponds to an energy maximum on the energy profile associated with the rotation around *T*1 determined by *ab initio* calculations. The quasi-planarity of the mol­ecule observed in the crystallographic structure could be encouraged by the formation of chalcogen bonds stabilizing this conformation of p-bSe.

## Database survey

**Figure 10:**
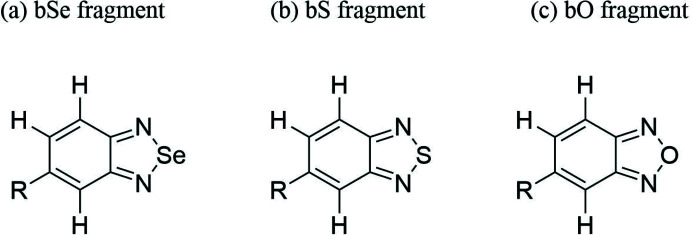
Fragments searched for in the CSD during the database survey analysis: (*a*) bSe fragment (*b*) bS fragment (*c*) bO fragment.

The Cambridge Structural Database (CSD version 5.42; Groom *et al.*, 2016 ▸) was searched with *ConQuest* (version 2021.2.0; Bruno *et al.*, 2002 ▸) for benzoselena­diazole, benzo­thia­diazole and benzoxa­diazole fragments (Fig. 10 ▸). The search for the benzoselena­diazole fragment resulted in 57 hits. Chalcogen bonds are observed in all of these hits. In particular, chalcogen–π inter­actions are observed in four structures [CSD refcodes: QIBQUQ (Lee *et al.*, 2018 ▸), VOPMEV (Lee *et al.*, 2019 ▸), VOPNAS (Lee *et al.*, 2019 ▸), and YIWLOG (Tan *et al.*, 2008 ▸)], listed in Table 2 ▸. The search for a benzo­thia­diazole fragment resulted in 34 hits. Chalcogen bonds but no chalcogen–π inter­actions are observed in these hits. The search for a benzoxa­diazole fragment resulted in 24 hits but no chalcogen inter­actions are observed with the oxygen atom of benzoxa­diazole as a chalcogen donor.

## Synthesis and crystallization

The synthesis of the various compounds was reported by Kozlova *et al.* (2021*a* ▸). Crystallization of the 5-(1*H*-indol-3-yl)-benzotriazole derivatives were carried out by the solvent evaporation method. The compounds were dissolved in THF, chloro­form, di­chloro­methane or DMF until complete dissolution. Slow evaporation of the solvent at room temperature (293–297 K) yielded colorless crystals that were then picked for XRD analysis. Crystals of 5-(1*H*-indol-3-yl)-2,1,3-benzo­thia­diazole and 5-(1*H*-indol-3-yl)-2,1,3-benzoxa­diazole were obtained from chloro­form while the protected benzoselena­diazole (5-[1-(benzensulfon­yl)-1*H*-indol-3-yl]-2,1,3-benzo­sel­ena­diazole) was crystallized in THF.

## Refinement

Crystal data, data collection and structure refinement details are summarized in Table 3 ▸. In all of the structures, hydrogen atoms were placed in calculated positions and refined using a riding model [C—H bond length of 0.93 Å, with *U*_iso_(H) = 1.2*U*_eq_(C)]. In the structure of 5-(1*H*-indol-3-yl)-2,1,3-benzo­thia­diazole, bS, the hydrogen on the nitro­gen atom in the indole group was refined without constraint and the refined N—H distance is 0.78 (2) Å.

## Quantum *ab initio* methodology

All the mol­ecules investigated in the study (bS, bO, bSe, p-bSe) were optimized starting from the crystal coordinates using the density functional method (DFT) with the exchange-correlation functional ωB97XD and the 6-31+G* basis set. Because we were not able to crystallize the bSe compound, this mol­ecule was created by substitution of the sulfur atom for a selenium atom from the coordinates of the bS mol­ecule. The optimizations were performed with *Gaussian16a* (Frisch *et al.*, 2016 ▸) the in gas phase. The electrostatic potential was calculated from the SCF-type density and was sliced by making 80 cubic points evenly distributed on a rectangular grid automatically generated by *Gaussian16a*. The resulting maps were visualized using *DrawMol* (Liegeois, 2021 ▸). For the conformational scans, the optimized structures were analyzed using relaxed scans around the torsion angle (*T*1) formed between the indole ring and the benzo­diazole part from 0 to 360° by steps of 20°. The resulting conformations close to an energy minimum were extracted and refined by a new optimization at the same level of approximation. The preparation of the input files, as well as the visualization of the results was performed with the *DrawMol* and *DrawSpectrum* suite of programs (Liegeois, 2021 ▸). The graphs were drawn with the program *Prism* from *GraphPad* (one-way ANOVA followed by Dunnetts multiple comparisons test, *Prism* version 8.0.0 for Windows; GraphPad, 2021 ▸).

## Supplementary Material

Crystal structure: contains datablock(s) p-bSe, bS, bO. DOI: 10.1107/S2056989022002948/vm2261sup1.cif

Structure factors: contains datablock(s) p-bSe. DOI: 10.1107/S2056989022002948/vm2261p-bSesup2.hkl

Structure factors: contains datablock(s) bS. DOI: 10.1107/S2056989022002948/vm2261bSsup3.hkl

Structure factors: contains datablock(s) bO. DOI: 10.1107/S2056989022002948/vm2261bOsup4.hkl

Supporting information file. DOI: 10.1107/S2056989022002948/vm2261p-bSesup5.cml

Supporting information file. DOI: 10.1107/S2056989022002948/vm2261bSsup6.cml

Supporting information file. DOI: 10.1107/S2056989022002948/vm2261bOsup7.cml

CCDC references: 2159479, 2159480, 2159481

Additional supporting information:  crystallographic information; 3D view; checkCIF report

## Figures and Tables

**Table 1:** Hydrogen-bond geometry (Å, °) for p-bSe[Chem scheme1]

*D*—H⋯*A*	*D*—H	H⋯*A*	*D*⋯*A*	*D*—H⋯*A*
C8—H8⋯O2^i^	0.93	2.46	3.380 (3)	168
C14—H14⋯O1	0.93	2.54	3.099 (4)	119

Symmetry code: (i) 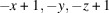 .

**Table 2:** Chalcogen bonds (Å, °) observed in benzoselena­diazole fragments in the CSD

CCDC refcode	Se label	Atoms of the π system	Se⋯centroid distance	N—Se⋯centroid angle
QIBQUQ*^*a*^*	Se1	C18–C23	3.3676 (7)	147.08 (4)
QIBQUQ*^*a*^*	Se1	C12–C17	3.0597 (8)	172.30 (5)
VOPMEV*^*b*^*	Se1	C5/C7/C10/C5*B*/C7*B*/C10*B*	3.8142 (3)	163.56 (4)
VOPNAS*^*c*^*	Se1	C1–C5/C10	3.802 (2)	162.5 (1)
VOPNAS*^*c*^*	Se2	C1–C5/C10	3.654 (2)	166.5 (1)
YIWLOG*^*d*^*	Se5	C10–C15	4.032 (3)	144.6 (2)
YIWLOG*^*d*^*	Se6	C34–C39	4.232 (4)	164.3 (4)

Notes: (*a*) Lee *et al.* (2018 ▸); (*b*) Lee *et al.*, 2019 ▸); (*c*) Lee *et al.*, 2019 ▸); (*d*) Tan *et al.* (2008 ▸).

**Table 3:** Experimental details

	p-bSe	bS	bO
Crystal data			
Chemical formula	C_20_H_13_N_3_O_2_SSe	C_14_H_9_N_3_S	C_14_H_9_N_3_O
*M*_r_	438.35	251.30	235.24
Crystal system, space group	Triclinic, *P* 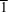	Orthorhombic, *P* *b* *c* *a*	Orthorhombic, *P* *b* *c* *a*
Temperature (K)	295	295	295
*a*, *b*, *c* (Å)	7.7760 (3), 9.9573 (4), 11.4124 (6)	7.5884 (1), 7.1060 (1), 43.2464 (7)	12.0256 (7), 7.7396 (5), 23.8551 (16)
α, β, γ (°)	90.970 (4), 92.771 (4), 94.283 (3)	90, 90, 90	90, 90, 90
*V* (Å^3^)	879.95 (7)	2331.98 (6)	2220.3 (2)
*Z*	2	8	8
Radiation type	Cu *K*α	Cu *K*α	Cu *K*α
μ (mm^−1^)	4.18	2.32	0.75
Crystal size (mm)	0.19 × 0.10 × 0.01	0.29 × 0.18 × 0.04	0.12 × 0.09 × 0.03

Data collection			
Diffractometer	Xcalibur, Ruby, Gemini ultra	Xcalibur, Ruby, Gemini ultra R	Xcalibur, Ruby, Gemini ultra
Absorption correction	Analytical (*CrysAlis PRO*; Rigaku OD, 2020 ▸)	Analytical (*CrysAlis PRO*; Rigaku OD, 2020 ▸)	Analytical (*CrysAlis PRO*; Rigaku OD, 2020 ▸)
*T*_min_, *T*_max_	0.663, 0.958	0.663, 0.920	0.941, 0.981
No. of measured, independent and observed [*I* > 2σ(*I*)] reflections	9941, 3113, 2571	10906, 2072, 1825	6883, 1972, 1275
*R*_int_	0.030	0.025	0.047
(sin θ/λ)_max_ (Å^−1^)	0.598	0.598	0.597

Refinement			
*R*[*F* ^2^ > 2σ(*F* ^2^)], *wR*(*F* ^2^), *S*	0.037, 0.101, 1.04	0.035, 0.098, 1.07	0.050, 0.134, 1.06
No. of reflections	3113	2072	1972
No. of parameters	244	167	166
H-atom treatment	H-atom parameters constrained	H atoms treated by a mixture of independent and constrained refinement	H atoms treated by a mixture of independent and constrained refinement
Δρ_max_, Δρ_min_ (e Å^−3^)	0.46, −0.61	0.20, −0.30	0.14, −0.16

Computer programs: *CrysAlis PRO* (Rigaku OD, 2020 ▸), *SHELXT2014/5* (Sheldrick, 2015*a* ▸), *SHELXL2016/6* (Sheldrick, 2015*b* ▸), *ShelXle* (Hübschle *et al.*, 2011 ▸), *OLEX2* (Dolomanov *et al.*, 2009 ▸), and*Mercury* (Macrae *et al.*, 2020 ▸).

## supplementary crystallographic information

### 5-[1-(Benzenesulfonyl)-1H-indol-3-yl]-2,1,3-benzoselenadiazole (p-bSe) Crystal data

**Table:**

C_20_H_13_N_3_O_2_SSe	*Z* = 2
*M**_r_* = 438.35	*F*(000) = 440
Triclinic, *P*1	*D*_x_ = 1.654 Mg m^−^^3^
*a* = 7.7760 (3) Å	Cu *K*α radiation, λ = 1.54184 Å
*b* = 9.9573 (4) Å	Cell parameters from 4183 reflections
*c* = 11.4124 (6) Å	θ = 3.9–66.7°
α = 90.970 (4)°	µ = 4.18 mm^−^^1^
β = 92.771 (4)°	*T* = 295 K
γ = 94.283 (3)°	Plate, colourless
*V* = 879.95 (7) Å^3^	0.19 × 0.10 × 0.01 mm

### 5-[1-(Benzenesulfonyl)-1H-indol-3-yl]-2,1,3-benzoselenadiazole (p-bSe) Data collection

**Table:**

Xcalibur, Ruby, Gemini ultra diffractometer	3113 independent reflections
Radiation source: fine-focus sealed X-ray tube, Enhance Ultra (Cu) X-ray Source	2571 reflections with *I* > 2σ(*I*)
Detector resolution: 5.1856 pixels mm^-1^	*R*_int_ = 0.030
ω scans	θ_max_ = 67.2°, θ_min_ = 3.9°
Absorption correction: analytical (CrysAlisPro; Rigaku OD, 2020)	*h* = −9→9
*T*_min_ = 0.663, *T*_max_ = 0.958	*k* = −11→9
9941 measured reflections	*l* = −13→13

### 5-[1-(Benzenesulfonyl)-1H-indol-3-yl]-2,1,3-benzoselenadiazole (p-bSe) Refinement

**Table:**

Refinement on *F*^2^	Primary atom site location: dual
Least-squares matrix: full	Secondary atom site location: dual
*R*[*F*^2^ > 2σ(*F*^2^)] = 0.037	Hydrogen site location: inferred from neighbouring sites
*wR*(*F*^2^) = 0.101	H-atom parameters constrained
*S* = 1.04	*w* = 1/[σ^2^(*F*_o_^2^) + (0.0588*P*)^2^ + 0.2071*P*] where *P* = (*F*_o_^2^ + 2*F*_c_^2^)/3
3113 reflections	(Δ/σ)_max_ = 0.001
244 parameters	Δρ_max_ = 0.46 e Å^−^^3^
0 restraints	Δρ_min_ = −0.61 e Å^−^^3^

### 5-[1-(Benzenesulfonyl)-1H-indol-3-yl]-2,1,3-benzoselenadiazole (p-bSe) Special details

**Table:**

Geometry. All esds (except the esd in the dihedral angle between two l.s. planes) are estimated using the full covariance matrix. The cell esds are taken into account individually in the estimation of esds in distances, angles and torsion angles; correlations between esds in cell parameters are only used when they are defined by crystal symmetry. An approximate (isotropic) treatment of cell esds is used for estimating esds involving l.s. planes.
Refinement. Structures were solved by the dual-space method of ShelXT (Sheldrick, 2015a) within Olex^2^ (Dolomanov *et al.*, 2009). Structures were refined by the least- squares method implemented in SHELXL (Sheldrick, 2015b) within ShelXle (Hübschle *et al.*, 2011). Structures and crystal packings were visualized using Mercury (Macrae *et al.*, 2020).

### 5-[1-(Benzenesulfonyl)-1H-indol-3-yl]-2,1,3-benzoselenadiazole (p-bSe) Fractional atomic coordinates and isotropic or equivalent isotropic displacement parameters (Å^2^)

**Table:**

	*x*	*y*	*z*	*U*_iso_*/*U*_eq_	
Se1	0.90079 (4)	0.81216 (3)	0.75959 (3)	0.07181 (16)	
S1	0.52981 (9)	0.00138 (6)	0.25117 (6)	0.05354 (19)	
O1	0.4385 (3)	−0.0082 (2)	0.13980 (18)	0.0639 (5)	
O2	0.4503 (3)	−0.0486 (2)	0.35371 (18)	0.0650 (5)	
N1	0.9080 (3)	0.7558 (3)	0.6120 (2)	0.0674 (7)	
N2	0.7790 (4)	0.6657 (3)	0.8062 (2)	0.0685 (7)	
N3	0.5808 (3)	0.1632 (2)	0.2811 (2)	0.0539 (5)	
C1	0.8223 (3)	0.6341 (3)	0.6050 (2)	0.0529 (6)	
C2	0.7984 (4)	0.5541 (3)	0.5005 (2)	0.0558 (6)	
H2	0.846444	0.585254	0.432073	0.067*	
C3	0.7060 (3)	0.4321 (2)	0.4993 (2)	0.0481 (6)	
C4	0.6317 (4)	0.3868 (3)	0.6070 (2)	0.0570 (7)	
H4	0.566610	0.304496	0.606199	0.068*	
C5	0.6530 (4)	0.4592 (3)	0.7089 (3)	0.0620 (7)	
H5	0.604349	0.426314	0.776555	0.074*	
C6	0.7506 (4)	0.5862 (3)	0.7115 (2)	0.0553 (6)	
C7	0.6741 (3)	0.3458 (2)	0.3939 (2)	0.0477 (6)	
C8	0.6014 (3)	0.2176 (3)	0.3945 (2)	0.0519 (6)	
H8	0.569709	0.172160	0.461603	0.062*	
C9	0.6494 (3)	0.2603 (3)	0.2037 (2)	0.0517 (6)	
C10	0.7064 (3)	0.3750 (3)	0.2714 (2)	0.0503 (6)	
C11	0.7763 (5)	0.4873 (3)	0.2134 (3)	0.0675 (8)	
H11	0.814269	0.565849	0.255173	0.081*	
C12	0.7881 (5)	0.4801 (3)	0.0936 (3)	0.0802 (10)	
H12	0.834370	0.554638	0.054712	0.096*	
C13	0.7326 (5)	0.3643 (3)	0.0297 (3)	0.0730 (9)	
H13	0.744430	0.362173	−0.050976	0.088*	
C14	0.6602 (4)	0.2520 (3)	0.0831 (2)	0.0616 (7)	
H14	0.620702	0.174545	0.040267	0.074*	
C15	0.7292 (4)	−0.0687 (2)	0.2387 (2)	0.0545 (6)	
C16	0.8268 (4)	−0.0925 (3)	0.3403 (3)	0.0637 (7)	
H16	0.787011	−0.072995	0.413787	0.076*	
C17	0.9851 (4)	−0.1459 (3)	0.3296 (3)	0.0732 (9)	
H17	1.052470	−0.162969	0.396443	0.088*	
C18	1.0426 (4)	−0.1736 (3)	0.2205 (3)	0.0733 (9)	
H18	1.149596	−0.208273	0.214087	0.088*	
C19	0.9442 (5)	−0.1509 (3)	0.1210 (3)	0.0723 (8)	
H19	0.984606	−0.170801	0.047749	0.087*	
C20	0.7853 (4)	−0.0986 (3)	0.1287 (3)	0.0636 (7)	
H20	0.717547	−0.083800	0.061463	0.076*	

### 5-[1-(Benzenesulfonyl)-1H-indol-3-yl]-2,1,3-benzoselenadiazole (p-bSe) Atomic displacement parameters (Å^2^)

**Table:**

	*U*^11^	*U*^22^	*U*^33^	*U*^12^	*U*^13^	*U*^23^
Se1	0.0753 (2)	0.0682 (2)	0.0690 (3)	−0.00422 (16)	−0.00371 (17)	−0.02183 (16)
S1	0.0587 (4)	0.0467 (4)	0.0525 (4)	−0.0097 (3)	−0.0021 (3)	−0.0040 (3)
O1	0.0655 (12)	0.0654 (12)	0.0575 (12)	−0.0055 (9)	−0.0107 (9)	−0.0115 (9)
O2	0.0722 (12)	0.0598 (11)	0.0604 (12)	−0.0146 (9)	0.0078 (10)	0.0011 (9)
N1	0.0683 (15)	0.0635 (15)	0.0673 (16)	−0.0116 (11)	0.0024 (12)	−0.0126 (12)
N2	0.0805 (17)	0.0695 (16)	0.0551 (15)	0.0090 (13)	−0.0007 (13)	−0.0112 (12)
N3	0.0681 (14)	0.0440 (11)	0.0473 (12)	−0.0086 (9)	0.0006 (10)	−0.0024 (9)
C1	0.0520 (14)	0.0522 (14)	0.0534 (16)	0.0035 (11)	−0.0050 (12)	−0.0072 (11)
C2	0.0604 (16)	0.0571 (15)	0.0486 (15)	−0.0046 (12)	0.0043 (12)	−0.0038 (11)
C3	0.0519 (14)	0.0454 (13)	0.0471 (14)	0.0061 (10)	−0.0019 (11)	−0.0009 (10)
C4	0.0734 (18)	0.0461 (14)	0.0511 (16)	0.0018 (12)	0.0011 (13)	0.0019 (11)
C5	0.081 (2)	0.0578 (16)	0.0477 (16)	0.0068 (14)	0.0042 (14)	0.0048 (12)
C6	0.0607 (16)	0.0553 (15)	0.0501 (15)	0.0136 (12)	−0.0058 (13)	−0.0059 (12)
C7	0.0507 (14)	0.0481 (13)	0.0440 (14)	0.0034 (10)	0.0012 (11)	−0.0017 (10)
C8	0.0604 (15)	0.0502 (14)	0.0443 (14)	−0.0010 (11)	0.0010 (12)	0.0005 (11)
C9	0.0562 (15)	0.0506 (14)	0.0477 (15)	0.0020 (11)	−0.0008 (12)	0.0019 (11)
C10	0.0567 (15)	0.0463 (14)	0.0483 (15)	0.0048 (11)	0.0041 (12)	0.0004 (11)
C11	0.096 (2)	0.0478 (15)	0.0566 (18)	−0.0091 (14)	0.0058 (16)	−0.0007 (12)
C12	0.120 (3)	0.0613 (18)	0.0573 (19)	−0.0120 (18)	0.0133 (18)	0.0073 (14)
C13	0.103 (2)	0.0705 (19)	0.0446 (16)	−0.0002 (16)	0.0070 (16)	0.0032 (13)
C14	0.0788 (19)	0.0597 (16)	0.0450 (15)	0.0000 (14)	−0.0025 (14)	−0.0026 (12)
C15	0.0639 (16)	0.0401 (13)	0.0568 (16)	−0.0098 (11)	−0.0022 (13)	−0.0009 (11)
C16	0.0712 (19)	0.0583 (16)	0.0591 (17)	−0.0064 (13)	−0.0054 (14)	0.0028 (13)
C17	0.072 (2)	0.0647 (19)	0.080 (2)	−0.0023 (15)	−0.0186 (17)	0.0078 (16)
C18	0.0690 (19)	0.0574 (18)	0.093 (3)	0.0020 (14)	0.0006 (18)	0.0007 (16)
C19	0.082 (2)	0.0645 (19)	0.071 (2)	0.0070 (16)	0.0059 (17)	−0.0070 (15)
C20	0.0748 (19)	0.0565 (16)	0.0581 (18)	0.0004 (13)	−0.0013 (15)	−0.0041 (13)

### 5-[1-(Benzenesulfonyl)-1H-indol-3-yl]-2,1,3-benzoselenadiazole (p-bSe) Geometric parameters (Å, º)

**Table:**

Se1—N1	1.772 (3)	C8—H8	0.9300
Se1—N2	1.782 (3)	C9—C14	1.384 (4)
S1—O1	1.424 (2)	C9—C10	1.399 (4)
S1—O2	1.430 (2)	C10—C11	1.398 (4)
S1—N3	1.657 (2)	C11—C12	1.376 (5)
S1—C15	1.758 (3)	C11—H11	0.9300
N1—C1	1.337 (4)	C12—C13	1.383 (5)
N2—C6	1.329 (4)	C12—H12	0.9300
N3—C8	1.391 (3)	C13—C14	1.379 (4)
N3—C9	1.414 (4)	C13—H13	0.9300
C1—C2	1.420 (4)	C14—H14	0.9300
C1—C6	1.436 (4)	C15—C20	1.383 (4)
C2—C3	1.365 (4)	C15—C16	1.388 (4)
C2—H2	0.9300	C16—C17	1.386 (5)
C3—C4	1.448 (4)	C16—H16	0.9300
C3—C7	1.466 (4)	C17—C18	1.374 (5)
C4—C5	1.355 (4)	C17—H17	0.9300
C4—H4	0.9300	C18—C19	1.370 (5)
C5—C6	1.424 (4)	C18—H18	0.9300
C5—H5	0.9300	C19—C20	1.383 (5)
C7—C8	1.357 (4)	C19—H19	0.9300
C7—C10	1.463 (4)	C20—H20	0.9300
			
N1—Se1—N2	95.07 (11)	C14—C9—C10	123.6 (3)
O1—S1—O2	120.66 (12)	C14—C9—N3	129.3 (3)
O1—S1—N3	107.53 (12)	C10—C9—N3	107.1 (2)
O2—S1—N3	104.65 (12)	C11—C10—C9	117.9 (3)
O1—S1—C15	108.75 (13)	C11—C10—C7	134.3 (3)
O2—S1—C15	109.34 (13)	C9—C10—C7	107.8 (2)
N3—S1—C15	104.72 (12)	C12—C11—C10	119.0 (3)
C1—N1—Se1	106.3 (2)	C12—C11—H11	120.5
C6—N2—Se1	105.8 (2)	C10—C11—H11	120.5
C8—N3—C9	108.0 (2)	C11—C12—C13	121.5 (3)
C8—N3—S1	123.64 (18)	C11—C12—H12	119.3
C9—N3—S1	126.7 (2)	C13—C12—H12	119.3
N1—C1—C2	124.0 (3)	C14—C13—C12	121.4 (3)
N1—C1—C6	116.0 (3)	C14—C13—H13	119.3
C2—C1—C6	120.0 (2)	C12—C13—H13	119.3
C3—C2—C1	120.8 (3)	C13—C14—C9	116.6 (3)
C3—C2—H2	119.6	C13—C14—H14	121.7
C1—C2—H2	119.6	C9—C14—H14	121.7
C2—C3—C4	118.3 (2)	C20—C15—C16	121.6 (3)
C2—C3—C7	123.5 (2)	C20—C15—S1	119.6 (2)
C4—C3—C7	118.2 (2)	C16—C15—S1	118.8 (2)
C5—C4—C3	122.8 (3)	C17—C16—C15	118.4 (3)
C5—C4—H4	118.6	C17—C16—H16	120.8
C3—C4—H4	118.6	C15—C16—H16	120.8
C4—C5—C6	119.4 (3)	C18—C17—C16	120.2 (3)
C4—C5—H5	120.3	C18—C17—H17	119.9
C6—C5—H5	120.3	C16—C17—H17	119.9
N2—C6—C5	124.5 (3)	C19—C18—C17	120.8 (3)
N2—C6—C1	116.8 (3)	C19—C18—H18	119.6
C5—C6—C1	118.7 (2)	C17—C18—H18	119.6
C8—C7—C10	106.2 (2)	C18—C19—C20	120.4 (3)
C8—C7—C3	123.9 (2)	C18—C19—H19	119.8
C10—C7—C3	129.8 (2)	C20—C19—H19	119.8
C7—C8—N3	110.8 (2)	C19—C20—C15	118.6 (3)
C7—C8—H8	124.6	C19—C20—H20	120.7
N3—C8—H8	124.6	C15—C20—H20	120.7
			
N2—Se1—N1—C1	−0.2 (2)	C8—N3—C9—C14	179.1 (3)
N1—Se1—N2—C6	−0.4 (2)	S1—N3—C9—C14	13.3 (4)
O1—S1—N3—C8	151.5 (2)	C8—N3—C9—C10	−1.8 (3)
O2—S1—N3—C8	22.0 (3)	S1—N3—C9—C10	−167.7 (2)
C15—S1—N3—C8	−93.0 (2)	C14—C9—C10—C11	0.7 (4)
O1—S1—N3—C9	−44.8 (3)	N3—C9—C10—C11	−178.4 (3)
O2—S1—N3—C9	−174.2 (2)	C14—C9—C10—C7	−179.9 (3)
C15—S1—N3—C9	70.8 (3)	N3—C9—C10—C7	1.0 (3)
Se1—N1—C1—C2	−180.0 (2)	C8—C7—C10—C11	179.5 (3)
Se1—N1—C1—C6	0.7 (3)	C3—C7—C10—C11	0.8 (5)
N1—C1—C2—C3	−178.5 (3)	C8—C7—C10—C9	0.3 (3)
C6—C1—C2—C3	0.8 (4)	C3—C7—C10—C9	−178.5 (3)
C1—C2—C3—C4	0.5 (4)	C9—C10—C11—C12	−0.8 (5)
C1—C2—C3—C7	178.9 (2)	C7—C10—C11—C12	180.0 (3)
C2—C3—C4—C5	−1.3 (4)	C10—C11—C12—C13	−0.1 (6)
C7—C3—C4—C5	−179.8 (3)	C11—C12—C13—C14	1.2 (6)
C3—C4—C5—C6	0.7 (4)	C12—C13—C14—C9	−1.3 (5)
Se1—N2—C6—C5	−178.2 (2)	C10—C9—C14—C13	0.3 (5)
Se1—N2—C6—C1	0.9 (3)	N3—C9—C14—C13	179.3 (3)
C4—C5—C6—N2	179.7 (3)	O1—S1—C15—C20	11.5 (3)
C4—C5—C6—C1	0.6 (4)	O2—S1—C15—C20	145.2 (2)
N1—C1—C6—N2	−1.2 (4)	N3—S1—C15—C20	−103.2 (2)
C2—C1—C6—N2	179.5 (3)	O1—S1—C15—C16	−168.6 (2)
N1—C1—C6—C5	178.0 (3)	O2—S1—C15—C16	−35.0 (2)
C2—C1—C6—C5	−1.4 (4)	N3—S1—C15—C16	76.7 (2)
C2—C3—C7—C8	171.4 (3)	C20—C15—C16—C17	0.8 (4)
C4—C3—C7—C8	−10.2 (4)	S1—C15—C16—C17	−179.0 (2)
C2—C3—C7—C10	−10.1 (4)	C15—C16—C17—C18	0.3 (4)
C4—C3—C7—C10	168.3 (3)	C16—C17—C18—C19	−0.9 (5)
C10—C7—C8—N3	−1.4 (3)	C17—C18—C19—C20	0.4 (5)
C3—C7—C8—N3	177.4 (2)	C18—C19—C20—C15	0.6 (5)
C9—N3—C8—C7	2.1 (3)	C16—C15—C20—C19	−1.3 (4)
S1—N3—C8—C7	168.4 (2)	S1—C15—C20—C19	178.6 (2)

### 5-[1-(Benzenesulfonyl)-1H-indol-3-yl]-2,1,3-benzoselenadiazole (p-bSe) Hydrogen-bond geometry (Å, º)

**Table:**

*D*—H···*A*	*D*—H	H···*A*	*D*···*A*	*D*—H···*A*
C8—H8···O2^i^	0.93	2.46	3.380 (3)	168
C14—H14···O1	0.93	2.54	3.099 (4)	119

Symmetry code: (i) −*x*+1, −*y*, −*z*+1.

### 5-[1-(Benzenesulfonyl)-1H-indol-3-yl]-2,1,3-benzothiadiazole (bS) Crystal data

**Table:**

C_14_H_9_N_3_S	*D*_x_ = 1.432 Mg m^−^^3^
*M**_r_* = 251.30	Cu *K*α radiation, λ = 1.54184 Å
Orthorhombic, *P**b**c**a*	Cell parameters from 4620 reflections
*a* = 7.5884 (1) Å	θ = 4.1–67.1°
*b* = 7.1060 (1) Å	µ = 2.32 mm^−^^1^
*c* = 43.2464 (7) Å	*T* = 295 K
*V* = 2331.98 (6) Å^3^	Plate, colourless
*Z* = 8	0.29 × 0.18 × 0.04 mm
*F*(000) = 1040	

### 5-[1-(Benzenesulfonyl)-1H-indol-3-yl]-2,1,3-benzothiadiazole (bS) Data collection

**Table:**

Xcalibur, Ruby, Gemini ultra R diffractometer	2072 independent reflections
Radiation source: fine-focus sealed tube	1825 reflections with *I* > 2σ(*I*)
Detector resolution: 5.1856 pixels mm^-1^	*R*_int_ = 0.025
ω scans	θ_max_ = 67.2°, θ_min_ = 4.1°
Absorption correction: analytical (CrysAlisPro; Rigaku OD, 2020)	*h* = −9→8
*T*_min_ = 0.663, *T*_max_ = 0.920	*k* = −8→7
10906 measured reflections	*l* = −51→49

### 5-[1-(Benzenesulfonyl)-1H-indol-3-yl]-2,1,3-benzothiadiazole (bS) Refinement

**Table:**

Refinement on *F*^2^	Primary atom site location: dual
Least-squares matrix: full	Secondary atom site location: dual
*R*[*F*^2^ > 2σ(*F*^2^)] = 0.035	Hydrogen site location: mixed
*wR*(*F*^2^) = 0.098	H atoms treated by a mixture of independent and constrained refinement
*S* = 1.07	*w* = 1/[σ^2^(*F*_o_^2^) + (0.0478*P*)^2^ + 0.7413*P*] where *P* = (*F*_o_^2^ + 2*F*_c_^2^)/3
2072 reflections	(Δ/σ)_max_ = 0.001
167 parameters	Δρ_max_ = 0.20 e Å^−^^3^
0 restraints	Δρ_min_ = −0.30 e Å^−^^3^

### 5-[1-(Benzenesulfonyl)-1H-indol-3-yl]-2,1,3-benzothiadiazole (bS) Special details

**Table:**

Geometry. All esds (except the esd in the dihedral angle between two l.s. planes) are estimated using the full covariance matrix. The cell esds are taken into account individually in the estimation of esds in distances, angles and torsion angles; correlations between esds in cell parameters are only used when they are defined by crystal symmetry. An approximate (isotropic) treatment of cell esds is used for estimating esds involving l.s. planes.
Refinement. Structures were solved by the dual-space method of ShelXT (Sheldrick, 2015a) within Olex^2^ (Dolomanov *et al.*, 2009). Structures were refined by the least- squares method implemented in SHELXL (Sheldrick, 2015b) within ShelXle (Hübschle *et al.*, 2011). Structures and crystal packings were visualized using Mercury (Macrae *et al.*, 2020).

### 5-[1-(Benzenesulfonyl)-1H-indol-3-yl]-2,1,3-benzothiadiazole (bS) Fractional atomic coordinates and isotropic or equivalent isotropic displacement parameters (Å^2^)

**Table:**

	*x*	*y*	*z*	*U*_iso_*/*U*_eq_	
S1	1.08880 (6)	0.65005 (9)	0.46249 (2)	0.0684 (2)	
N1	1.05463 (19)	0.6759 (2)	0.42591 (3)	0.0539 (4)	
N3	0.2880 (2)	0.8454 (2)	0.33487 (4)	0.0567 (4)	
H3N	0.206 (3)	0.904 (3)	0.3295 (5)	0.065 (6)*	
C9	0.5346 (2)	0.6723 (2)	0.33531 (4)	0.0369 (3)	
C5	0.6078 (2)	0.7131 (2)	0.39422 (3)	0.0384 (4)	
C6	0.7877 (2)	0.7053 (2)	0.39392 (3)	0.0396 (4)	
H6	0.849325	0.714886	0.375400	0.048*	
C7	0.5027 (2)	0.7429 (2)	0.36611 (4)	0.0395 (4)	
N2	0.8896 (2)	0.6472 (3)	0.47547 (4)	0.0649 (5)	
C14	0.6590 (2)	0.5510 (2)	0.32215 (4)	0.0453 (4)	
H14	0.750683	0.502873	0.334031	0.054*	
C10	0.3973 (2)	0.7415 (2)	0.31639 (4)	0.0441 (4)	
C1	0.8784 (2)	0.6826 (2)	0.42215 (4)	0.0415 (4)	
C4	0.5157 (2)	0.6949 (3)	0.42314 (4)	0.0480 (4)	
H4	0.393193	0.698955	0.422964	0.058*	
C2	0.7846 (2)	0.6662 (3)	0.45063 (4)	0.0476 (4)	
C3	0.5985 (2)	0.6722 (3)	0.45054 (4)	0.0543 (5)	
H3	0.534867	0.660972	0.468820	0.065*	
C13	0.6439 (3)	0.5037 (3)	0.29140 (4)	0.0567 (5)	
H13	0.725749	0.422248	0.282623	0.068*	
C11	0.3836 (3)	0.6946 (3)	0.28513 (4)	0.0547 (5)	
H11	0.293096	0.742288	0.272936	0.066*	
C8	0.3506 (2)	0.8457 (3)	0.36445 (4)	0.0518 (4)	
H8	0.297698	0.906861	0.381037	0.062*	
C12	0.5077 (3)	0.5761 (3)	0.27307 (4)	0.0594 (5)	
H12	0.501492	0.542837	0.252302	0.071*	

### 5-[1-(Benzenesulfonyl)-1H-indol-3-yl]-2,1,3-benzothiadiazole (bS) Atomic displacement parameters (Å^2^)

**Table:**

	*U*^11^	*U*^22^	*U*^33^	*U*^12^	*U*^13^	*U*^23^
S1	0.0416 (3)	0.1158 (5)	0.0479 (3)	−0.0007 (3)	−0.00962 (19)	−0.0036 (3)
N1	0.0354 (7)	0.0777 (11)	0.0486 (8)	−0.0024 (7)	−0.0023 (6)	−0.0024 (7)
N3	0.0500 (9)	0.0557 (9)	0.0645 (10)	0.0187 (8)	−0.0130 (7)	0.0043 (7)
C9	0.0349 (8)	0.0340 (7)	0.0417 (8)	−0.0034 (6)	−0.0024 (6)	0.0053 (6)
C5	0.0371 (8)	0.0380 (8)	0.0399 (8)	−0.0005 (6)	0.0003 (6)	−0.0026 (6)
C6	0.0363 (8)	0.0460 (9)	0.0365 (8)	0.0000 (7)	0.0031 (6)	−0.0003 (6)
C7	0.0363 (8)	0.0375 (8)	0.0448 (8)	0.0013 (7)	−0.0009 (6)	0.0014 (7)
N2	0.0484 (9)	0.1062 (14)	0.0401 (8)	0.0008 (9)	−0.0042 (7)	−0.0029 (8)
C14	0.0431 (9)	0.0462 (9)	0.0466 (9)	0.0039 (7)	−0.0008 (7)	0.0019 (7)
C10	0.0456 (9)	0.0373 (8)	0.0494 (9)	−0.0009 (7)	−0.0065 (7)	0.0082 (7)
C1	0.0336 (8)	0.0490 (9)	0.0421 (8)	−0.0013 (7)	0.0012 (6)	−0.0035 (7)
C4	0.0323 (8)	0.0651 (11)	0.0465 (9)	0.0003 (8)	0.0048 (7)	−0.0020 (8)
C2	0.0423 (9)	0.0634 (11)	0.0370 (8)	0.0001 (8)	−0.0005 (7)	−0.0032 (7)
C3	0.0424 (10)	0.0812 (13)	0.0393 (9)	−0.0010 (9)	0.0090 (7)	−0.0006 (8)
C13	0.0638 (12)	0.0558 (11)	0.0504 (10)	0.0032 (9)	0.0050 (9)	−0.0068 (8)
C11	0.0603 (11)	0.0553 (10)	0.0486 (10)	−0.0060 (9)	−0.0165 (8)	0.0119 (8)
C8	0.0473 (10)	0.0537 (10)	0.0543 (10)	0.0137 (8)	−0.0026 (8)	−0.0043 (8)
C12	0.0739 (13)	0.0635 (12)	0.0409 (9)	−0.0103 (11)	−0.0051 (9)	−0.0008 (8)

### 5-[1-(Benzenesulfonyl)-1H-indol-3-yl]-2,1,3-benzothiadiazole (bS) Geometric parameters (Å, º)

**Table:**

S1—N2	1.6128 (17)	N2—C2	1.344 (2)
S1—N1	1.6136 (16)	C14—C13	1.376 (2)
N1—C1	1.348 (2)	C14—H14	0.9300
N3—C8	1.365 (2)	C10—C11	1.396 (2)
N3—C10	1.368 (2)	C1—C2	1.428 (2)
N3—H3N	0.79 (2)	C4—C3	1.351 (2)
C9—C14	1.399 (2)	C4—H4	0.9300
C9—C10	1.413 (2)	C2—C3	1.413 (3)
C9—C7	1.444 (2)	C3—H3	0.9300
C5—C6	1.367 (2)	C13—C12	1.400 (3)
C5—C4	1.438 (2)	C13—H13	0.9300
C5—C7	1.469 (2)	C11—C12	1.366 (3)
C6—C1	1.411 (2)	C11—H11	0.9300
C6—H6	0.9300	C8—H8	0.9300
C7—C8	1.368 (2)	C12—H12	0.9300
			
N2—S1—N1	101.07 (8)	N1—C1—C6	126.36 (15)
C1—N1—S1	106.37 (12)	N1—C1—C2	112.81 (15)
C8—N3—C10	109.73 (15)	C6—C1—C2	120.82 (15)
C8—N3—H3N	123.5 (16)	C3—C4—C5	123.18 (16)
C10—N3—H3N	126.6 (16)	C3—C4—H4	118.4
C14—C9—C10	118.42 (15)	C5—C4—H4	118.4
C14—C9—C7	134.62 (15)	N2—C2—C3	126.70 (17)
C10—C9—C7	106.86 (14)	N2—C2—C1	113.72 (16)
C6—C5—C4	119.35 (15)	C3—C2—C1	119.58 (15)
C6—C5—C7	122.68 (14)	C4—C3—C2	118.11 (16)
C4—C5—C7	117.97 (14)	C4—C3—H3	120.9
C5—C6—C1	118.95 (14)	C2—C3—H3	120.9
C5—C6—H6	120.5	C14—C13—C12	121.24 (18)
C1—C6—H6	120.5	C14—C13—H13	119.4
C8—C7—C9	106.16 (14)	C12—C13—H13	119.4
C8—C7—C5	125.33 (15)	C12—C11—C10	117.74 (17)
C9—C7—C5	128.51 (14)	C12—C11—H11	121.1
C2—N2—S1	106.03 (13)	C10—C11—H11	121.1
C13—C14—C9	119.16 (16)	N3—C8—C7	110.01 (16)
C13—C14—H14	120.4	N3—C8—H8	125.0
C9—C14—H14	120.4	C7—C8—H8	125.0
N3—C10—C11	130.51 (16)	C11—C12—C13	121.26 (17)
N3—C10—C9	107.23 (15)	C11—C12—H12	119.4
C11—C10—C9	122.17 (16)	C13—C12—H12	119.4
			
N2—S1—N1—C1	−0.35 (15)	C5—C6—C1—N1	−178.66 (17)
C4—C5—C6—C1	−1.0 (2)	C5—C6—C1—C2	0.5 (2)
C7—C5—C6—C1	177.87 (14)	C6—C5—C4—C3	0.8 (3)
C14—C9—C7—C8	−175.18 (18)	C7—C5—C4—C3	−178.16 (17)
C10—C9—C7—C8	0.92 (18)	S1—N2—C2—C3	−179.70 (18)
C14—C9—C7—C5	4.5 (3)	S1—N2—C2—C1	−0.2 (2)
C10—C9—C7—C5	−179.43 (15)	N1—C1—C2—N2	−0.1 (2)
C6—C5—C7—C8	−143.47 (18)	C6—C1—C2—N2	−179.37 (16)
C4—C5—C7—C8	35.4 (2)	N1—C1—C2—C3	179.49 (18)
C6—C5—C7—C9	36.9 (3)	C6—C1—C2—C3	0.2 (3)
C4—C5—C7—C9	−144.19 (17)	C5—C4—C3—C2	0.0 (3)
N1—S1—N2—C2	0.31 (16)	N2—C2—C3—C4	179.05 (19)
C10—C9—C14—C13	0.2 (2)	C1—C2—C3—C4	−0.4 (3)
C7—C9—C14—C13	175.93 (18)	C9—C14—C13—C12	0.6 (3)
C8—N3—C10—C11	176.87 (18)	N3—C10—C11—C12	−175.41 (19)
C8—N3—C10—C9	0.3 (2)	C9—C10—C11—C12	0.7 (3)
C14—C9—C10—N3	176.06 (14)	C10—N3—C8—C7	0.3 (2)
C7—C9—C10—N3	−0.78 (18)	C9—C7—C8—N3	−0.7 (2)
C14—C9—C10—C11	−0.8 (2)	C5—C7—C8—N3	179.60 (15)
C7—C9—C10—C11	−177.65 (15)	C10—C11—C12—C13	0.1 (3)
S1—N1—C1—C6	179.53 (14)	C14—C13—C12—C11	−0.7 (3)
S1—N1—C1—C2	0.27 (19)		

### 5-(1H-Indol-3-yl)-2,1,3-benzoxadiazole (bO) Crystal data

**Table:**

C_14_H_9_N_3_O	*D*_x_ = 1.407 Mg m^−^^3^
*M**_r_* = 235.24	Cu *K*α radiation, λ = 1.54184 Å
Orthorhombic, *P**b**c**a*	Cell parameters from 1395 reflections
*a* = 12.0256 (7) Å	θ = 7.4–66.3°
*b* = 7.7396 (5) Å	µ = 0.75 mm^−^^1^
*c* = 23.8551 (16) Å	*T* = 295 K
*V* = 2220.3 (2) Å^3^	Plate, clear yellow
*Z* = 8	0.12 × 0.09 × 0.03 mm
*F*(000) = 976	

### 5-(1H-Indol-3-yl)-2,1,3-benzoxadiazole (bO) Data collection

**Table:**

Xcalibur, Ruby, Gemini ultra diffractometer	1972 independent reflections
Radiation source: fine-focus sealed X-ray tube, Enhance Ultra (Cu) X-ray Source	1275 reflections with *I* > 2σ(*I*)
Detector resolution: 5.1856 pixels mm^-1^	*R*_int_ = 0.047
ω scans	θ_max_ = 67.1°, θ_min_ = 3.7°
Absorption correction: analytical (CrysAlisPro; Rigaku OD, 2020)	*h* = −14→12
*T*_min_ = 0.941, *T*_max_ = 0.981	*k* = −6→9
6883 measured reflections	*l* = −28→19

### 5-(1H-Indol-3-yl)-2,1,3-benzoxadiazole (bO) Refinement

**Table:**

Refinement on *F*^2^	Primary atom site location: dual
Least-squares matrix: full	Secondary atom site location: dual
*R*[*F*^2^ > 2σ(*F*^2^)] = 0.050	Hydrogen site location: mixed
*wR*(*F*^2^) = 0.134	H atoms treated by a mixture of independent and constrained refinement
*S* = 1.06	*w* = 1/[σ^2^(*F*_o_^2^) + (0.0633*P*)^2^ + 0.0873*P*] where *P* = (*F*_o_^2^ + 2*F*_c_^2^)/3
1972 reflections	(Δ/σ)_max_ < 0.001
166 parameters	Δρ_max_ = 0.14 e Å^−^^3^
0 restraints	Δρ_min_ = −0.16 e Å^−^^3^

### 5-(1H-Indol-3-yl)-2,1,3-benzoxadiazole (bO) Special details

**Table:**

Geometry. All esds (except the esd in the dihedral angle between two l.s. planes) are estimated using the full covariance matrix. The cell esds are taken into account individually in the estimation of esds in distances, angles and torsion angles; correlations between esds in cell parameters are only used when they are defined by crystal symmetry. An approximate (isotropic) treatment of cell esds is used for estimating esds involving l.s. planes.
Refinement. Structures were solved by the dual-space method of ShelXT (Sheldrick, 2015a) within Olex^2^ (Dolomanov *et al.*, 2009). Structures were refined by the least- squares method implemented in SHELXL (Sheldrick, 2015b) within ShelXle (Hübschle *et al.*, 2011). Structures and crystal packings were visualized using Mercury (Macrae *et al.*, 2020).

### 5-(1H-Indol-3-yl)-2,1,3-benzoxadiazole (bO) Fractional atomic coordinates and isotropic or equivalent isotropic displacement parameters (Å^2^)

**Table:**

	*x*	*y*	*z*	*U*_iso_*/*U*_eq_	
O1	0.26211 (19)	0.2142 (3)	0.43917 (8)	0.0909 (6)	
N1	0.1692 (2)	0.2567 (3)	0.46968 (9)	0.0801 (7)	
N2	0.3589 (2)	0.2507 (3)	0.46824 (9)	0.0822 (7)	
N3	0.52478 (16)	0.6406 (3)	0.70573 (9)	0.0640 (6)	
H3N	0.581 (2)	0.696 (3)	0.7137 (11)	0.077*	
C1	0.2075 (2)	0.3199 (3)	0.51701 (10)	0.0629 (6)	
C2	0.3259 (2)	0.3161 (3)	0.51611 (10)	0.0616 (6)	
C3	0.3883 (2)	0.3735 (3)	0.56268 (10)	0.0610 (6)	
H3A	0.465597	0.368956	0.562224	0.073*	
C4	0.33330 (18)	0.4356 (3)	0.60815 (9)	0.0520 (6)	
C5	0.21290 (18)	0.4396 (3)	0.60788 (10)	0.0566 (6)	
H5	0.176549	0.482253	0.639392	0.068*	
C6	0.1509 (2)	0.3849 (3)	0.56443 (10)	0.0658 (7)	
H6	0.073675	0.389643	0.565567	0.079*	
C7	0.39274 (17)	0.5038 (3)	0.65679 (9)	0.0506 (5)	
C8	0.49217 (18)	0.5924 (3)	0.65382 (10)	0.0610 (6)	
H8	0.530990	0.615615	0.620952	0.073*	
C9	0.36413 (16)	0.4987 (2)	0.71516 (9)	0.0473 (5)	
C10	0.44927 (17)	0.5855 (3)	0.74484 (10)	0.0513 (6)	
C11	0.4497 (2)	0.6039 (3)	0.80242 (11)	0.0624 (6)	
H11	0.506433	0.663393	0.820582	0.075*	
C12	0.3635 (2)	0.5313 (3)	0.83195 (11)	0.0654 (7)	
H12	0.361933	0.541208	0.870791	0.078*	
C13	0.27815 (19)	0.4427 (3)	0.80449 (11)	0.0606 (6)	
H13	0.220822	0.394500	0.825468	0.073*	
C14	0.27709 (17)	0.4252 (3)	0.74691 (10)	0.0527 (5)	
H14	0.219735	0.365798	0.729246	0.063*	

### 5-(1H-Indol-3-yl)-2,1,3-benzoxadiazole (bO) Atomic displacement parameters (Å^2^)

**Table:**

	*U*^11^	*U*^22^	*U*^33^	*U*^12^	*U*^13^	*U*^23^
O1	0.1045 (15)	0.1109 (16)	0.0571 (10)	−0.0033 (12)	−0.0023 (11)	−0.0055 (10)
N1	0.0843 (15)	0.0991 (17)	0.0569 (14)	−0.0107 (13)	−0.0056 (13)	0.0038 (12)
N2	0.0843 (16)	0.1040 (17)	0.0582 (14)	0.0035 (13)	0.0010 (12)	−0.0021 (12)
N3	0.0455 (10)	0.0674 (13)	0.0791 (15)	−0.0127 (9)	−0.0027 (10)	0.0060 (11)
C1	0.0670 (15)	0.0709 (15)	0.0507 (15)	−0.0074 (12)	−0.0041 (12)	0.0099 (12)
C2	0.0682 (15)	0.0693 (15)	0.0473 (14)	0.0029 (12)	0.0075 (12)	0.0066 (12)
C3	0.0510 (13)	0.0737 (15)	0.0585 (15)	0.0027 (12)	0.0037 (12)	0.0057 (12)
C4	0.0474 (11)	0.0533 (12)	0.0552 (13)	−0.0006 (10)	0.0027 (11)	0.0089 (11)
C5	0.0466 (12)	0.0683 (14)	0.0547 (14)	0.0018 (11)	0.0013 (11)	0.0035 (11)
C6	0.0506 (13)	0.0811 (17)	0.0655 (16)	−0.0038 (12)	−0.0020 (12)	0.0079 (13)
C7	0.0437 (11)	0.0512 (11)	0.0570 (14)	0.0000 (10)	−0.0030 (10)	0.0048 (11)
C8	0.0485 (12)	0.0708 (14)	0.0636 (15)	−0.0056 (11)	0.0031 (11)	0.0118 (12)
C9	0.0414 (10)	0.0439 (10)	0.0567 (13)	0.0029 (9)	0.0002 (10)	0.0028 (10)
C10	0.0440 (11)	0.0474 (12)	0.0626 (15)	0.0012 (10)	−0.0038 (11)	0.0010 (11)
C11	0.0602 (14)	0.0566 (13)	0.0704 (16)	0.0025 (12)	−0.0105 (13)	−0.0099 (12)
C12	0.0720 (16)	0.0630 (15)	0.0613 (15)	0.0119 (13)	0.0023 (13)	−0.0066 (12)
C13	0.0551 (13)	0.0618 (14)	0.0648 (15)	0.0076 (11)	0.0122 (12)	0.0040 (12)
C14	0.0424 (11)	0.0514 (12)	0.0642 (14)	0.0018 (10)	0.0023 (11)	0.0031 (11)

### 5-(1H-Indol-3-yl)-2,1,3-benzoxadiazole (bO) Geometric parameters (Å, º)

**Table:**

O1—N1	1.373 (3)	C5—H5	0.9300
O1—N2	1.384 (3)	C6—H6	0.9300
N1—C1	1.314 (3)	C7—C8	1.380 (3)
N2—C2	1.310 (3)	C7—C9	1.435 (3)
N3—C8	1.351 (3)	C8—H8	0.9300
N3—C10	1.370 (3)	C9—C14	1.411 (3)
N3—H3N	0.83 (3)	C9—C10	1.415 (3)
C1—C6	1.413 (3)	C10—C11	1.381 (3)
C1—C2	1.424 (4)	C11—C12	1.374 (3)
C2—C3	1.413 (3)	C11—H11	0.9300
C3—C4	1.358 (3)	C12—C13	1.397 (4)
C3—H3A	0.9300	C12—H12	0.9300
C4—C5	1.448 (3)	C13—C14	1.380 (3)
C4—C7	1.461 (3)	C13—H13	0.9300
C5—C6	1.345 (3)	C14—H14	0.9300
			
N1—O1—N2	111.69 (18)	C8—C7—C9	105.7 (2)
C1—N1—O1	105.1 (2)	C8—C7—C4	124.2 (2)
C2—N2—O1	105.1 (2)	C9—C7—C4	130.03 (19)
C8—N3—C10	110.23 (19)	N3—C8—C7	110.0 (2)
C8—N3—H3N	126.4 (19)	N3—C8—H8	125.0
C10—N3—H3N	123.3 (19)	C7—C8—H8	125.0
N1—C1—C6	130.7 (2)	C14—C9—C10	117.4 (2)
N1—C1—C2	109.2 (2)	C14—C9—C7	135.2 (2)
C6—C1—C2	120.1 (2)	C10—C9—C7	107.39 (18)
N2—C2—C3	130.2 (2)	N3—C10—C11	130.0 (2)
N2—C2—C1	108.9 (2)	N3—C10—C9	106.7 (2)
C3—C2—C1	120.8 (2)	C11—C10—C9	123.3 (2)
C4—C3—C2	118.7 (2)	C12—C11—C10	117.7 (2)
C4—C3—H3A	120.6	C12—C11—H11	121.1
C2—C3—H3A	120.6	C10—C11—H11	121.1
C3—C4—C5	119.4 (2)	C11—C12—C13	121.0 (2)
C3—C4—C7	121.6 (2)	C11—C12—H12	119.5
C5—C4—C7	119.0 (2)	C13—C12—H12	119.5
C6—C5—C4	123.4 (2)	C14—C13—C12	121.4 (2)
C6—C5—H5	118.3	C14—C13—H13	119.3
C4—C5—H5	118.3	C12—C13—H13	119.3
C5—C6—C1	117.5 (2)	C13—C14—C9	119.2 (2)
C5—C6—H6	121.2	C13—C14—H14	120.4
C1—C6—H6	121.2	C9—C14—H14	120.4
			
N2—O1—N1—C1	0.4 (3)	C5—C4—C7—C9	35.3 (3)
N1—O1—N2—C2	−0.4 (3)	C10—N3—C8—C7	0.3 (3)
O1—N1—C1—C6	179.9 (3)	C9—C7—C8—N3	−0.1 (2)
O1—N1—C1—C2	−0.3 (3)	C4—C7—C8—N3	179.06 (19)
O1—N2—C2—C3	178.7 (2)	C8—C7—C9—C14	−177.6 (2)
O1—N2—C2—C1	0.2 (3)	C4—C7—C9—C14	3.3 (4)
N1—C1—C2—N2	0.0 (3)	C8—C7—C9—C10	−0.1 (2)
C6—C1—C2—N2	179.9 (2)	C4—C7—C9—C10	−179.2 (2)
N1—C1—C2—C3	−178.6 (2)	C8—N3—C10—C11	179.1 (2)
C6—C1—C2—C3	1.2 (3)	C8—N3—C10—C9	−0.4 (2)
N2—C2—C3—C4	−179.5 (3)	C14—C9—C10—N3	178.34 (18)
C1—C2—C3—C4	−1.1 (3)	C7—C9—C10—N3	0.3 (2)
C2—C3—C4—C5	0.6 (3)	C14—C9—C10—C11	−1.2 (3)
C2—C3—C4—C7	−177.4 (2)	C7—C9—C10—C11	−179.3 (2)
C3—C4—C5—C6	−0.1 (3)	N3—C10—C11—C12	−178.4 (2)
C7—C4—C5—C6	177.9 (2)	C9—C10—C11—C12	1.0 (3)
C4—C5—C6—C1	0.2 (3)	C10—C11—C12—C13	−0.3 (3)
N1—C1—C6—C5	179.0 (3)	C11—C12—C13—C14	−0.1 (3)
C2—C1—C6—C5	−0.8 (3)	C12—C13—C14—C9	−0.1 (3)
C3—C4—C7—C8	34.3 (3)	C10—C9—C14—C13	0.7 (3)
C5—C4—C7—C8	−143.6 (2)	C7—C9—C14—C13	178.0 (2)
C3—C4—C7—C9	−146.7 (2)		
